# *R*-α-Lipoic Acid and 4-Phenylbutyric Acid Have Distinct Hypolipidemic Mechanisms in Hepatic Cells

**DOI:** 10.3390/biomedicines8080289

**Published:** 2020-08-15

**Authors:** Bo He, Régis Moreau

**Affiliations:** Department of Nutrition and Health Sciences, University of Nebraska-Lincoln, Lincoln, NE 68583-0806, USA; bhe@huskers.unl.edu

**Keywords:** thioctic acid, butyrate, hyperlipidemia, dyslipidemia, triacylglycerides, cAMP, CPT1A, PCSK9, LDL receptor, HDAC inhibitor

## Abstract

The constitutive activation of the mechanistic target of rapamycin complex 1 (mTORC1) leads to the overproduction of apoB-containing triacylglycerol-rich lipoproteins in HepG2 cells. *R*-α-lipoic acid (LA) and 4-phenylbutyric acid (PBA) have hypolipidemic function but their mechanisms of action are not well understood. Here, we reported that LA and PBA regulate hepatocellular lipid metabolism via distinct mechanisms. The use of SQ22536, an inhibitor of adenylyl cyclase, revealed cAMP’s involvement in the upregulation of *CPT1A* expression by LA but not by PBA. LA decreased the secretion of proprotein convertase subtilisin/kexin type 9 (PCSK9) in the culture media of hepatic cells and increased the abundance of LDL receptor (LDLR) in cellular extracts in part through transcriptional upregulation. Although PBA induced *LDLR* gene expression, it did not translate into more LDLR proteins. PBA regulated cellular lipid homeostasis through the induction of *CPT1A* and *INSIG2* expression via an epigenetic mechanism involving the acetylation of histone H3, histone H4, and CBP-p300 at the *CPT1A* and *INSIG2* promoters.

## 1. Introduction

Although the association between LDL cholesterol (LDL-C) and coronary heart disease (CHD) risk is prominent in observational studies and clinical trials, the attention to elevated triacylglycerides (TAG) and low HDL cholesterol (HDL-C) has increased with respect to risk estimation and therapeutic targets for intervention [1]. Owing to the metabolic interconnection between TAG-rich lipoproteins, HDL and insulin resistance, hypertriglyceridemia is frequently observed in combination with low HDL-C and type 2 diabetes. In clinical trials, subjects with hypertriglyceridemia and an elevated LDL-C/HDL-C ratio have a disproportionately higher risk of CHD events [2,3]. Hypertriglyceridemia, which affects ~30% of U.S. adults [4], is defined as fasting blood TAG levels above 150 mg/dL (~1.8 mM) and according to the Adult Treatment Panel III guidelines, 150-199 mg/dL is considered borderline high, 200–499 mg/dL is considered high, and >500 mg/dL is considered very high [5]. Hepatic overproduction of VLDL is believed to be the main contributor to hypertriglyceridemia [6]. The accumulation of tissue TAG leads to increased synthesis and secretion of VLDL from the liver and impedes chylomicron (CM) hydrolysis due to competition of CM and VLDL in binding to lipoprotein lipase. This results in the delayed clearance of CM remnants within the circulation and raises blood TAG. It is critically important to identify new therapeutic strategies and dietary molecules capable of modulating TAG, VLDL and LDL metabolism simultaneously, and elucidate their regulatory points and mechanisms of action.

*R*-α-Lipoic acid (LA) is a naturally occurring enzyme cofactor synthesized from octanoic acid in most prokaryotic and eukaryotic microorganisms as well as plant and animal mitochondria, and plant plastids [7,8]. LA can also be absorbed from foods (leafy green vegetables and red meats) and dietary supplements. LA supplements, typically of 50 to 600 mg per dose, can raise the blood plasma concentrations of LA from 1 to 225 μM depending on the form (enantiomer composition, free acid or salt formulation), dose, and route of administration (oral or parenteral) [9,10]. Results from both animal and human studies indicated LA from nutritional supplements is safe [11] and readily bioavailable [10,12]. Upon ingestion of a dose of LA, its concentrations transiently increase in blood, liver, heart, skeletal muscle and most other tissues. This short-chain fatty acid (SCFA) has been shown to have a broad spectrum of biological functions, such as antioxidant activity [13], hypoglycemic action [14] and vasodilation effects [15]. LA regulates lipid metabolism by lowering blood and tissue TAG contents in various animal models and in some human studies [16]. Mechanistically, LA downregulates lipid synthesis at the transcriptional and post-translational levels, primarily in liver and white adipose tissue, while concomitantly upregulating markers of fatty acid oxidation in the liver and skeletal muscle [17,18,19,20,21]. Current evidence implicates carbohydrate-responsive element-binding protein (ChREBP), sterol regulatory element-binding protein-1c (SREBP1c), insulin-induced gene 2 (INSIG2), sirtuin 1 (SIRT1), AMP-activated protein kinase (AMPK), fibroblast growth factor 21 (FGF21), and peroxisome proliferator-activated receptor α (PPARα) in the mechanism of LA [22,23,24,25,26]. Despite some advances in elucidating the mechanism of action of LA, the direct molecular target of LA, which is quickly metabolized in cells, remains elusive. In particular, the notion that LA stimulates cAMP production has not been fully exploited in relation to lipid metabolism [27]. Past observations of LA’s catabolic properties may be explained by the cAMP/cAMP-responsive element-binding protein (CREB) signaling. In the present study, we investigated the relationship between LA and hepatic *CPT1A* in a cAMP-dependent manner.

4-Phenylbutyric acid (PBA) is an FDA-approved compound primarily used for the treatment of urea cycle disorders on the grounds that it scavenges ammonia [28]. This SCFA has been credited with chaperon-like properties, thus facilitating protein folding and trafficking, with the ability to inhibit histone deacetylation (HDAC inhibitor) [29]. Research showed that PBA was health-beneficial by lowering total cholesterol and TAG contents in the liver of a mouse model of hypothyroidism [30]. In a rat model of hepatic steatosis induced by high fructose feeding, PBA intervention significantly lowered TAG content, suppressed lipogenic enzyme acetyl-CoA carboxylase (ACC), fatty acid synthase (*FASN*) and stearoyl-CoA desaturase (SCD) expression and improved ER stress [31]. As an HDAC inhibitor, PBA is well studied in cancer research. In hepatocarcinoma Bel-7402 cells, PBA arrested cell cycle and enhanced the acetylation status of histone H4 [32]. Two studies in human glioblastoma reported that PBA delayed cell growth and proliferation and induced apoptosis [33,34]. Recently, another group showed that PBA was able to induce GLUT4 expression at both mRNA and protein levels in C2C12 myotubes by inhibiting HDAC5, and this response was independent of PBA acting as a chemical chaperone [35].

Although experimental evidence supports the TAG-lowering properties of LA and PBA, the molecular mechanisms by which these SCFA regulate hepatocellular VLDL metabolism and LDL clearance are unclear [36]. In recent years, genetic studies recognized the critical role of proprotein convertase subtilisin/kexin type 9 (PCSK9) in cholesterol homeostasis and its direct association with familial hypercholesterolemia and hypocholesterolemia [37,38]. Predominantly synthesized and secreted from the liver, PCSK9 binds to the LDL receptor (LDLR) on the cell surface and promotes the internalization and lysosomal degradation of LDLR thus lowering the capacity to uptake LDL particles from plasma. Consequently, suppressing PCSK9 has therapeutic potential in the fight against hyperlipidemia. Monoclonal antibodies have been developed as PCSK9 inhibitors, and two of these drugs are FDA-approved. However, the high costs and mode of delivery by subcutaneous injection limit their broad use, therefore, they are prescribed to high-risk individuals who cannot achieve their target LDL cholesterol with other pharmacotherapies. In this context, lowering PCSK9 through dietary molecules should be explored as a practical and cost-effective approach, and in this regard, LA showed encouraging potential. Feeding LA to Zucker Diabetic Fatty rats increased liver LDLR protein levels 2-fold, decreased plasma PCSK9 by 70%, and significantly lowered blood cholesterol [39].

**Figure 1:**
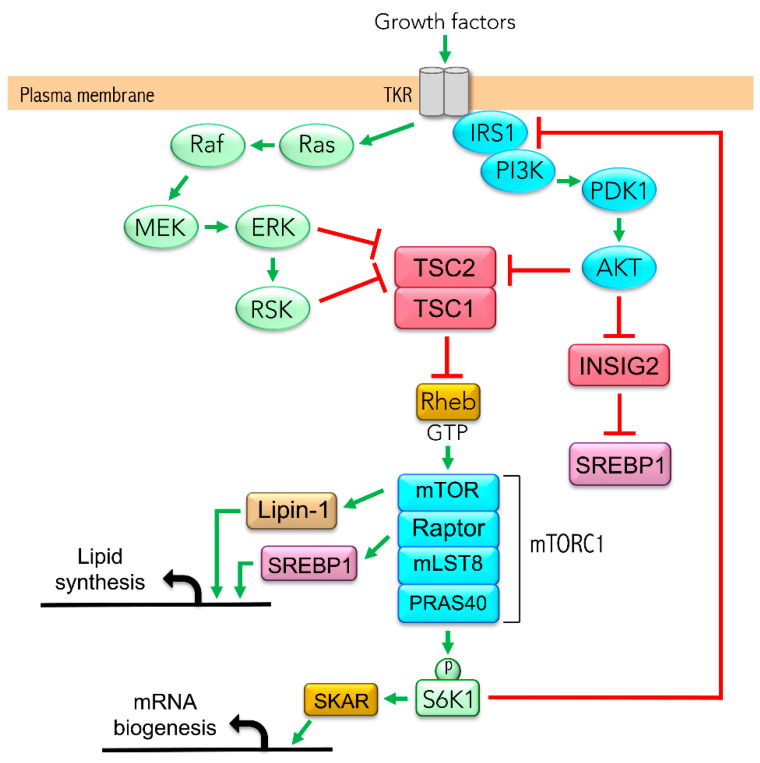
mTORC1 signaling involved in de novo lipogenesis and triacylglycerides (TAG) synthesis in hepatocyte. AKT, protein kinase B; ERK, extracellular signal-regulated kinase; INSIG2, insulin-induced gene 2 protein; IRS, insulin receptor substrate; Lipin-1, phosphatidate phosphatase LPIN1; MEK, mitogen-activated protein kinase; mLST8, mammalian lethal with SEC13 protein 8; mTOR, mechanistic target of rapamycin; PDK1, 3-phosphoinositide-dependent protein kinase 1; PI3K, phosphoinositide 3 kinase; PRAS40, proline-rich Akt substrate 40; RAF, proto-oncogene serine/threonine-protein kinase; Raptor, regulatory-associated protein of mTOR; Ras, GTPase Ras proteins; Rheb, Ras homolog enriched in the brain; RSK, p90 ribosomal protein S6 kinase; S6K1, p70 ribosomal protein S6 kinase B1; SKAR, S6K1 Aly/REF-like target; SREBP1, sterol regulatory element-binding protein 1; TKR, tyrosine kinase receptor; TSC1, hamartin; TSC2, tuberin.

Previous work from our group demonstrated that the hyperactivation of the mechanistic target of rapamycin complex 1 (mTORC1) leads to the overproduction of apoB-containing TAG-rich lipoproteins in HepG2 cells [40], a phenomenon ameliorated by LA and PBA. mTORC1, an evolutionarily conserved serine/threonine kinase, integrates signals coming from nutrients and growth factors to regulate protein, nucleotide and lipid syntheses. Growth factors regulate mTORC1 activity through the PI3K/Akt and/or Ras/MAPK signaling pathways, which converge on the tuberous sclerosis complex 1/2 (TSC1/2) to suppress its GTPase activity upon Rheb (Ras homolog enriched in brain) [41,42] (Figure 1). The deletion of either TSC1 or TSC2 increases the fractional GTP load of Rheb and activates mTORC1, whereas the elimination of scaffold protein Raptor abolishes mTORC1 activity. mTORC1 is required for insulin-mediated synthesis and processing of SREBP1 mRNA in rat hepatocytes [43]. In addition, mTORC1 activates SREBP1 through ribosomal protein S6 kinase beta-1 (p70S6K1), which phosphorylates lipin 1, thereby preventing its translocation to the nucleus and its inhibition of SREBP1 transcriptional activity [44]. However, the negative feedback inhibition of mTORC1 on the IRS/Akt pathway contributes to repressing lipid synthesis through Akt/INSIG2/SREBP1 when mTORC1 is constitutively active. The negative feedback is apparent in liver-specific *TSC1*-knockout mice, which are protected from diet-induced hepatic steatosis; they display impaired SREBP1 activity and lipogenesis [45,46], suggesting INSIG2 is a therapeutic target.

In the present study, we provided insight into the mechanisms by which LA and PBA counteract mTORC1-mediated lipid overload as it specifically pertains to genes of TAG and LDL metabolism. Our results show that both LA and PBA induced *CPT1A* and *INSIG2* expression in hepatic cells, notably under mTORC1 hyperactivation. The results also show cAMP’s involvement in the regulation of *CPT1A* expression by LA but not in that of PBA. In contrast, PBA induced *CPT1A* and *INSIG2* expression through epigenetic acetylation of CBP-p300 at the *CPT1A* and *INSIG2* promoters. The data support LA-mediated upregulation of LDLR receptor abundance through downregulation of PCSK9 secretion; no evidence of a similar mechanism was found for PBA.

## 2. Material and Methods

### 2.1. Reagents

*R*-α-lipoic acid (LA) from MAK Wood (#RALA1100134, Grafton, WI, USA); 4-Phenylbutyric acid (PBA) was from Acros Organics (#130,380,250, Thermo Fisher Scientific, Waltham, MA, USA); DMSO was from ATCC (4-X^™^, Manassas, VA, USA); and SQ22536 from Cayman Chemical (#13339, Ann Arbor, MI, USA). LA was dissolved in DMSO to a stock concentration of 200 mM and diluted to a final concentration of 200 μM. PBA was dissolved in DMEM medium to a final concentration of 8 mM. SQ22536 was dissolved in DMSO to a stock concentration of 50 mM and diluted to a final concentration of 100 μM. The final DMSO concentration did not exceed 0.2%.

### 2.2. Cell Culture

Stably transduced HepG2 and Huh-7 cell lines (HepG2-shScramble, HepG2-shRaptor, HepG2-shTSC2 and Huh7-shTSC2 cells) were created as previously described [40]. pLKO.1-Raptor (Addgene plasmid #1858) and pLKO.1 scramble shRNA (Addgene plasmid #1864) were from David Sabatini [47]. pLKO.1-TSC2 (Addgene plasmid #15478) was from Do-Hyung Kim [48]. HepG2 cells were cultured in DMEM (5.5 mM glucose, Gibco #11885076) supplemented with 10% (*v/v*) fetal bovine serum (FBS, low endotoxin, Gibco #16000044) and 0.25% antibiotics (Sigma-Aldrich #A5955). Huh-7 cells were cultured in DMEM (25 mM glucose, Gibco #11995040,) supplemented with 10% FBS and 1% (*v/v*) penicillin-streptomycin (ATCC #30-2300). Cells were cultured at 37 °C in 5% CO_2_ with medium change every 3–4 days. Prior to a treatment, cells were synchronized by withdrawing FBS overnight (16 h), and cultured in FBS-free DMEM containing 30 mM glucose to supply lipogenic substrates. Cells were treated with LA (200 μM) for 24 h, PBA (8 mM) for 6 h [40]. At these exposures, LA and PBA did not negatively affect cell viability or cause ER stress [40].

### 2.3. Trichloroacetic Acid (TCA) Precipitation of Proteins Secreted in Conditioned Media

Conditioned media were spun to remove cell debris. Equal volumes of spun media and 20% (*w/v*) TCA solution were mixed, vortexed, incubated on ice for 30 min and centrifuged (15,000× *g*, 10 min, 4 °C). The supernatant was carefully aspirated leaving the protein pellet to which 100 μL of cold acetone was added, vortexed and centrifuged (15,000× *g*, 10 min, 4 °C) to remove traces of TCA. After three acetone washes, the protein pellet was resuspended by sonication in Laemmli sample buffer and heat denatured (95 °C, 5 min) prior to Western blotting.

### 2.4. Whole Cell Lysate and Nuclear Fraction

To prepare whole cell lysate, cells were washed with cold PBS and scraped in RIPA buffer composed of 20 mM Tris-HCl, pH 7.5, 150 mM NaCl, 1 mM EDTA, 1 mM EGTA, 1% sodium deoxycholate, 1% NP-40, 1 mM DTT, and Halt protease/phosphatase inhibitors (Thermo Scientific). Total protein concentration of clarified supernatants was determined using the Pierce BCA Assay. To prepare nuclear fraction, cells were resuspended in a lysis buffer consisting of 10 mM Tris, pH 7.8, 1.5 mM MgCl_2_, 10 mM KCl, 0.5% NP-40, 1 mM DTT, and Halt protease/phosphatase inhibitors. Nuclei were pelleted by centrifugation (14,000× *g*, 15 min, 4 °C), resuspended in nuclear extract buffer (20 mM Tris, pH 7.8, 1.5 mM MgCl_2_, 0.42 M NaCl, 0.2 mM EDTA, 25% (*v/v)* glycerol, 1 mM DTT, Halt protease/phosphatase inhibitors), and sonicated. Cellular and nuclear proteins were resuspended in Laemmli sample buffer and heat denatured (95 °C, 5 min) prior to Western blotting.

### 2.5. Western Blotting

Proteins were resolved by reducing SDS–PAGE and transferred onto nitrocellulose membrane. The membranes were blocked with 5% milk in TBST for 1 hr and incubated with primary antibodies overnight. Antibodies against *CPT1A* (Abcam #Ab128568, Cambridge, MA, USA), LDLR (Santa Cruz Biotechnology #sc-18823, Dallas, TX, USA; Novus Biologicals #NBP1-06709, Centennial, CO, USA), PCSK9 (Santa Cruz Biotechnology #sc-515082), and β-actin (Sigma-Aldrich #A5441, St. Louis, MO, USA) were used. Blots were incubated with HRP-conjugated secondary antibodies and visualized using enhanced chemiluminescence (ECL, PerkinElmer, Shelton, CT, USA) on ProteinSimple imaging system. Band densitometry was determined with LI-COR Image Studio Lite software (Lincoln, NE, USA).

### 2.6. Quantitative Real-Time PCR (qRT-PCR)

Total RNA was isolated from cells using Bio-Rad Aurum^TM^ Total RNA mini kit (Bio-Rad #7326820, Hercules, CA), which includes an on-column DNase I treatment. First-strand cDNA was synthesized in the presence of oligo (dT) and random primers with iScript^TM^ Reverse Transcription Supermix (BioRad #1708841). qRT-PCR was performed on a BioRad CFX96 Real-Time PCR Detection System using Sso Advanced^™^ Universal SYBR^®^ Green Supermix (BioRad #1725272). Amplicon authenticity was confirmed by melt curve analysis and agarose gel electrophoresis. PCR efficiencies were assessed with serial dilutions of the template (0.0064–100 ng cDNA/reaction) and 0.3 μM of each primer, and by plotting the quantification cycle (Cq) vs. log amount of template. PCR efficiencies between target genes and housekeeping gene cyclophilin A (*PPIA*) were comparable, thus unknown amounts of target in the samples were determined relative to *PPIA*. For each primer pair, PCR controls included reaction containing total RNA instead of cDNA as template, or no template at all. Primer sequences are provided in Supplementary Table S1.

### 2.7. Chromatin Immunoprecipitation (ChIP) Assay

ChIP assays were performed as previously described [26]. Huh7-shTSC2 cells were crosslinked with 37% formaldehyde and chromatin was extracted. Samples were sonicated using Brandson SFX 550 to produce 250–700 bp chromatin fragments. After pre-clearing the sheared chromatin, samples were subjected to immunoprecipitation using the following the antibodies: anti-acetyl-CBP [Lys1535]/p300 [Lys1499] (Cell Signaling Technology #4771S, Danvers, MA, USA), anti-acetyl-histone H3 (Millipore #06–599, Burlington, MA, USA), anti-histone H3 (Millipore #07–690). Rabbit IgG was used as negative control and input (10%) was used as positive control. DNA was eluted and used for qRT-PCR analysis using the primers designed for the first intron of *CPT1A* and promoter region of *INSIG2* (Supplementary Table S2). Acetyl CBP/p300 occupancy in the first intron of *CPT1A* was normalized to input DNA, acetyl-histone H3 was normalized to histone H3 abundance and input DNA.

### 2.8. Statistical Analysis

Results are shown as means ± SEM. Statistical significance was determined by unpaired two-tailed Student’s *t*-test or one-way ANOVA followed by Tukey’s multiple comparisons test to detect differences between groups using GraphPad Prism software. All statistical tests were performed at the 5% significance level.

## 3. Results

### 3.1. LA and PBA Regulate Lipid Metabolism-Related Genes Irrespective of mTORC1 Activity Level

**Figure 2:**
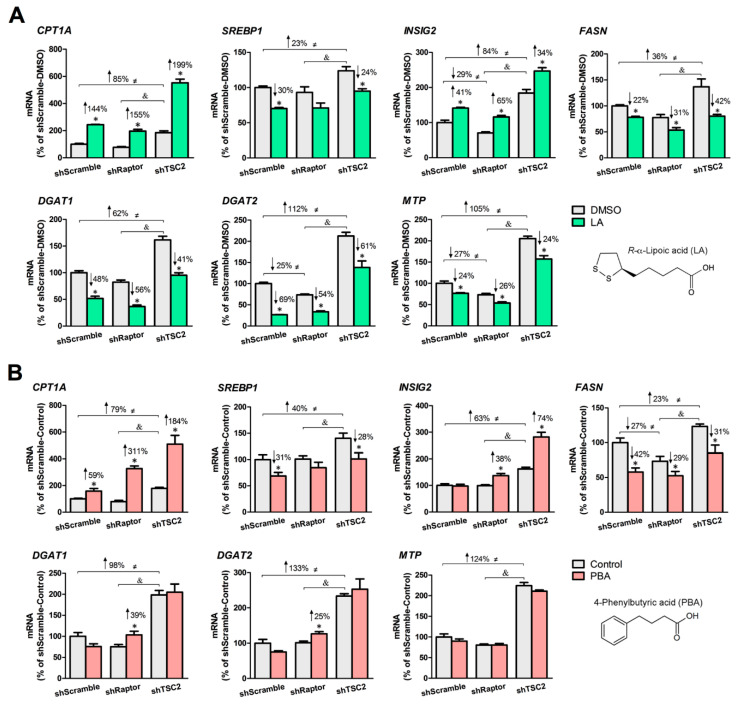
. *R*-α-lipoic acid (LA) and 4-phenylbutyric acid (PBA) upregulate the expression of lipolysis genes and downregulate that of genes involved in de novo lipogenesis, TAG synthesis and lipoprotein assembly in mTORC1-manipulated HepG2 cells. (**A**) LA (200 μM, 24 h) significantly stimulated the expression of *CPT1A* and *INSIG2* but decreased that of *SREBP1*, *FASN*, *DGAT1*, *DGAT2* and *MTP*. (**B**) PBA (8 mM, 6 h) significantly stimulated the expression of *CPT1A* and *INSIG2* but decreased that of *SREBP1* and *FASN*. * indicates a statistically significant effect of LA or PBA vs. vehicle control within a given stable HepG2 cell line; & indicates a statistically significant effect of shRaptor vs. shTSC2 vehicle control; ≠ indicates a statistically significant effect of mTORC1 between stable cell lines; Student’s *t*-test, *p* < 0.05, *n* = 4.

We created three stable HepG2 cell lines in which mTORC1 activity is normal (shScramble), low (shRaptor), or high (shTSC2) and demonstrated that application of LA or PBA in shTSC2 cells led to a significant lowering of VLDL-like particle secretion [40]. Herein, we examined the lipid-lowering properties of LA and PBA in all three HepG2 cell lines based on changes in expression of genes involved in lipolysis, lipogenesis, TAG synthesis, and lipoprotein assembly. Under nutrient-rich conditions (30 mM glucose), the basal level of lipid-related genes was higher (23–133%) in shTSC2 cells than in shScramble cells, whereas shRaptor cells trended to be lower comparing to shScramble cells (Figure 2A,B). In shScramble and shTSC2 cells, LA and PBA significantly decreased the expression of *SREBP1* (LA: −30% in shScramble cells, −24% in shTSC2 cells; PBA: −31% in shScramble cells, −28% in shTSC2 cells) and *FASN* (LA: −22% in shScramble cells, −50% in shTSC2 cells; PBA: −42% in shScramble cells, −64% in shTSC2 cells), and increased that of *CPT1A* (LA: +144% in shScramble cells, +199% in shTSC2 cells; PBA: +59% in shScramble cells, +184% in shTSC2 cells) and *INSIG2*, a negative regulator of *SREBP1* processing (LA: +41% in shScramble cells, +34% in shTSC2 cells; PBA: +74% in shTSC2 cells). These observations suggested that the two SCFAs cause a shift away from lipogenesis towards lipolysis irrespective of mTORC1 activity level; LA and PBA remained potent regulators of lipid metabolism even under constitutive active mTORC1. We noted a stronger response of LA (compared to PBA) toward genes involved in TAG synthesis and lipoprotein assembly. Specifically, LA significantly decreased mRNA levels of *DGAT1* (48% in shScramble cells, −41% in shTSC2 cells, −56% in shRaptor cells), *DGAT2* (−69% in shScramble cells, −61% in shTSC2 cells, −54% in shRaptor cells) and *MTP* (−24% in shScramble cells, −24% in shTSC2 cells, −26% in shRaptor cells). In contrast, PBA had no significant inhibitory effects on *DGAT1*, *DGAT2* and *MTP* expression in shScramble and shTSC2 cells, and rather increased the mRNA levels of *DGAT1* (+39%) and *DGAT2* (+25%) in shRaptor cells.

### 3.2. cAMP Mediates the Lipid-Lowering Action of LA

**Figure 3:**
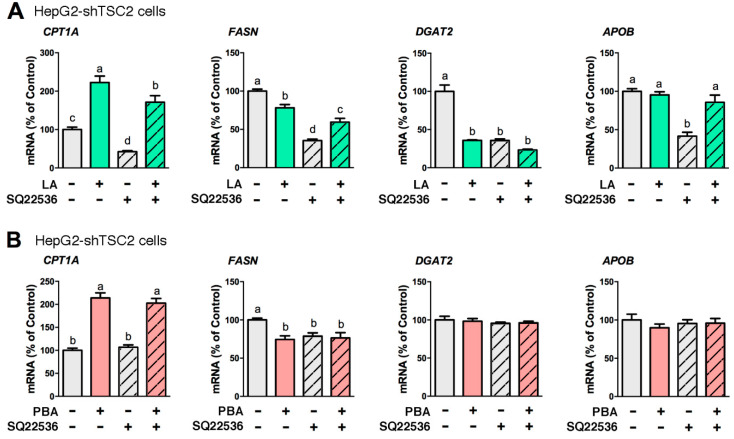
cAMP partakes in the mechanism of LA, but not in that of PBA. HepG2-shTSC2 cells were treated with either 200 μM LA for 24 h (**A**) or 8 mM PBA for 6 h (**B**) along with 100 μM SQ22536 or vehicle control. Total RNA was isolated and *CPT1A*, *FASN*, *DGAT2*, *APOB* mRNA levels quantified by qRT-PCR. Statistical significance was determined using one-way ANOVA followed by Tukey’s multiple comparisons test. Values not sharing a common letter are significantly different, *p* < 0.05, *n* = 6.

Generated from ATP by adenylyl cyclase within cell membranes, cAMP partakes in the regulation of lipid-related genes. Given LA and PBA modulate several lipid-related genes (*CPT1A*, *FASN*, *DGAT1* and *DGAT2*), we asked whether cAMP signaling was part of LA and PBA mechanisms of action. SQ22536, an inhibitor of adenylyl cyclase, significantly diminished LA-mediated stimulation of *CPT1A* expression in HepG2-shTSC2 cells revealing that cAMP mediated LA’s cellular signaling (Figure 3A). In contrast, PBA-mediated stimulation of *CPT1A* expression was unchanged in the presence or absence of SQ22536 (Figure 3B). Of note, SQ22536 alone repressed *FASN* and *DGAT2* expression (Figure 3).

### 3.3. PBA Acts as HDAC Inhibitor to Drive CPT1A and INSIG2 Expression

**Figure 4:**
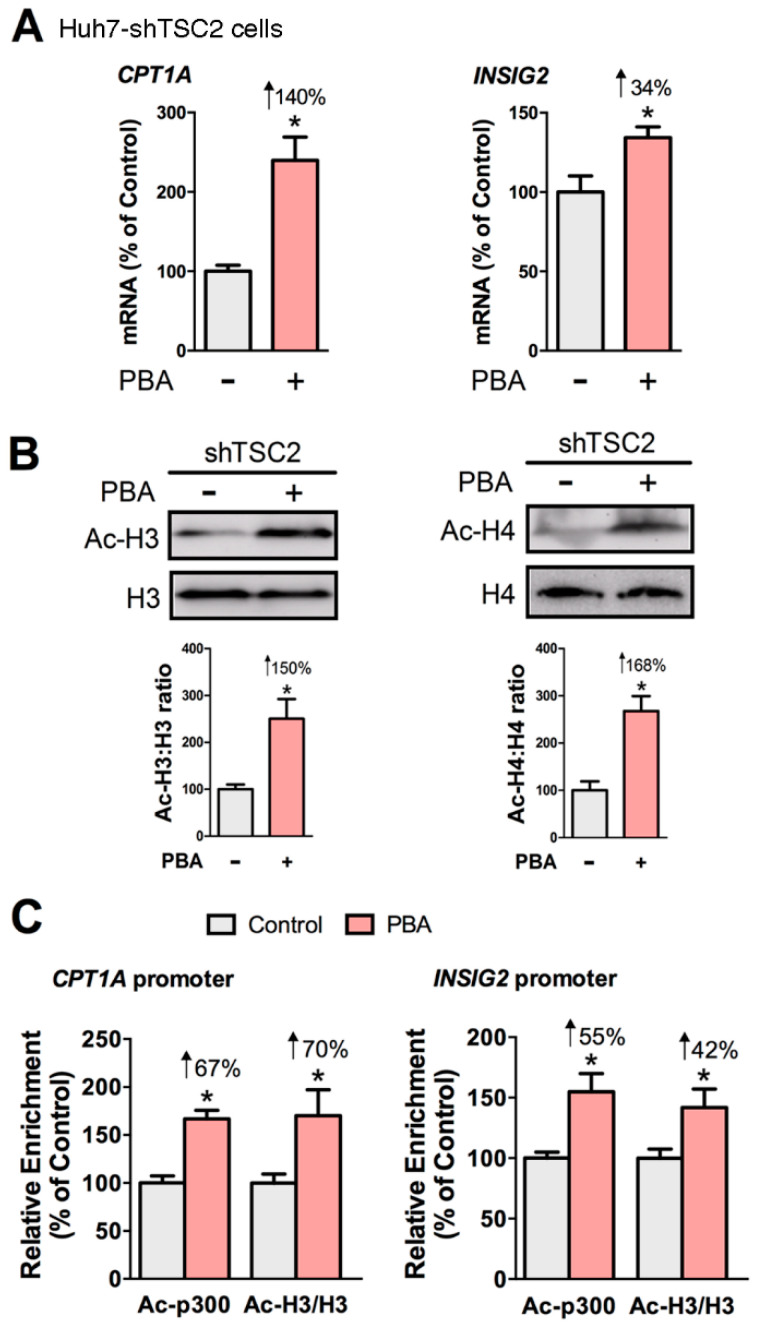
PBA enhances the acetylation of CBP/p300 and histone H3 at the *CPT1A* and *INSIG2* promoters in Huh7-shTSC2 cells. PBA (8 mM, 6 h) induced the gene expression of *CPT1A* and *INSIG2* (**A**), and the acetylation status of histone H3 and histone H4 (**B**). Chromatin immunoprecipitation (ChIP) assay revealed PBA-mediated enrichment of acetyl CBP/p300 (Ac-p300) and acetyl histone H3 (Ac-H3) at the *CPT1A* and *INSIG2* promoters (**C**). Statistical significance was determined using Student’s *t*-test, * *p* < 0.05, *n* = 4.

Since our results revealed that cAMP was not involved in the mechanism of PBA, we asked whether PBA acted as HDAC inhibitor to remodel chromatin and thus influence gene expression. To that end, we examined the gene expression of *CPT1A* and *INSIG2*, the acetylation of histones, and the abundance of acetylated CBP/p300 and acetylated histone H3 at the *CPT1A* and *INSIG2* promoters in Huh7-shTSC2 cells treated +/− PBA (8 mM, 6 h). The ChIP antibody we used detects CBP acetylated at Lys1535 or p300 acetylated at Lys1499. Acetylation of p300 at Lys1499 has been demonstrated to enhance p300 histone acetyltransferase (HAT) activity [49]. Results showed PBA significantly induced the expression of *CPT1A* and *INSIG2*, and the acetylation state of histones H3 and H4 (Figure 4A,B). ChIP data revealed that PBA caused a statistically significant enrichment of acetylated CBP/p300 (Ac-p300: +67% and +55%) and acetylated histone H3 (Ac-H3: +70% and +42%) at the *CPT1A* and *INSIG2* promoter regions indicating PBA modulated transcription through epigenetic modification (Figure 4C).

### 3.4. LA, But Not PBA, Suppresses PCSK9 Secretion

**Figure 5:**
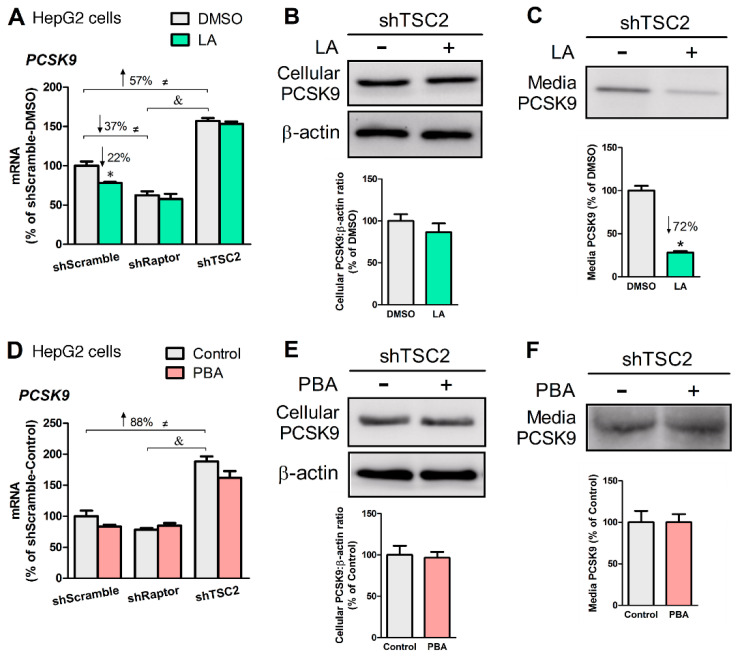
LA, but not PBA, repressed PCSK9 secretion. (**A**) *PCSK9* mRNA levels as affected by LA (200 μM, 24 h) in mTORC1-manipulated HepG2 cells. Cellular (**B**) and media (**C**) PCSK9 protein abundance from HepG2-shTSC2 treated +/− LA. (**D**) *PCSK9* mRNA levels as affected by PBA (8 mM, 6 h) in mTORC1-manipulated HepG2 cells. Cellular (**E**) and media (**F**) PCSK9 protein abundance in HepG2-shTSC2 treated +/− PBA. * indicates a statistically significant effect of LA vs. vehicle control within a given HepG2 stable cell line; & indicates a statistically significant effect of shRaptor vs. shTSC2 vehicle control; ≠ indicates a statistically significant effect of mTORC1 between stable cell lines; Student’s *t*-test, *p* < 0.05, *n* = 4.

Earlier studies demonstrated that LA feeding to rats significantly lowered plasma PCSK9 and enhanced hepatic LDLR [39]. These findings prompted us to examine whether LA and PBA might affect the LDLR signaling in an mTORC1-dependent manner. We found *PCSK9* mRNA levels correlated positively with mTORC1 activity in HepG2 cells (Figure 5A,D). LA (200 μM, 24 h) lowered *PCSK9* mRNA in shScramble cells (–22%, *p* < 0.05, Figure 5A) but did not in shTSC2 or shRaptor cells. However, LA did not significantly change *PCSK9* mRNA levels in shTSC2 cells, LA decreased PCSK9 abundance in media (–72%, *p* < 0.05, Figure 5C). PBA (8 mM, 6 h) did not affect the gene expression, media or cellular levels of PCSK9 (Figure 5D–F).

### 3.5. LA or PBA Induces LDLR Gene Expression, But Only LA Upregulates LDLR Protein

**Figure 6:**
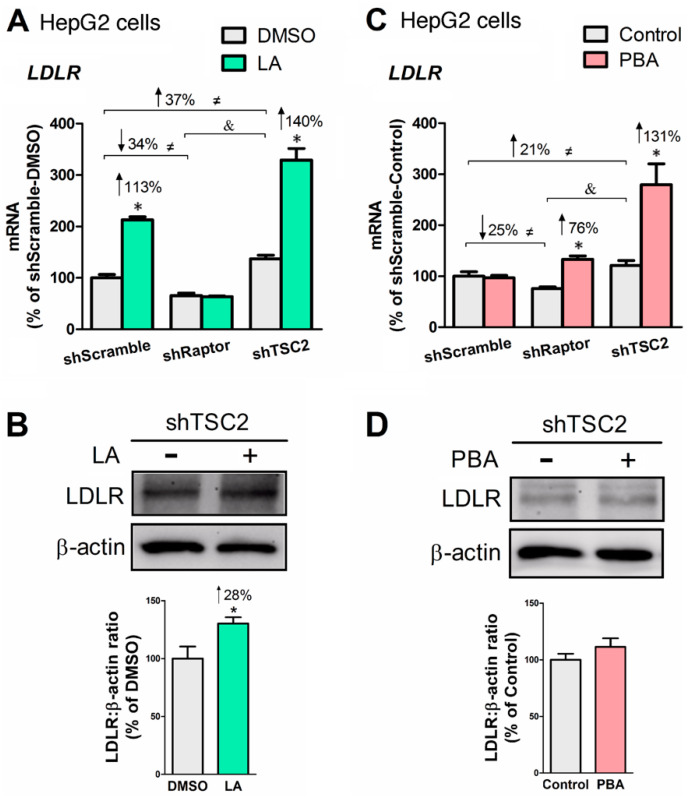
LA upregulated LDL receptor (LDLR) expression at both mRNA and protein levels, whereas PBA only increased *LDLR* mRNA. (**A**) *LDLR* mRNA levels as affected by LA in mTORC1-manipulated HepG2 cells. (**B**) LDLR protein abundance in HepG2-shTSC2 treated +/− LA. (**C**) *LDLR* mRNA level as affected by PBA in mTORC1-manipulated HepG2 cells. (**D**) LDLR protein abundance in HepG2-shTSC2 treated +/− PBA. * indicates a statistically significant effect of LA or PBA vs. vehicle control within a given cell line; & indicates a statistically significant effect of shRaptor vs. shTSC2 vehicle control; ≠ indicates a statistically significant effect of mTORC1 between stable cell lines; Student’s *t*-test, *p* < 0.05, *n* = 4.

Since PCSK9 negatively regulates LDL uptake by initiating LDL receptor (LDLR) degradation when binding to LDLR, we sought to determine LDLR status in liver cells treated +/− LA or +/− PBA. LA and PBA upregulated *LDLR* gene expression, and the effect was more pronounced in HepG2-shTSC2 cells (Figure 6A,C). Moreover, LA significantly increased cellular LDLR abundance (Figure 6B). Together, the data show that although LA and PBA upregulate *LDLR* gene expression, LA (but not PBA) upregulates LDLR protein abundance through suppression of PCSK9 secretion.

## 4. Discussion

The liver plays a critical role in lipid homeostasis and failure to maintain a balance may cause dyslipidemia in the form of hypertriglyceridemia, low high-density lipoprotein (HDL), or elevated small dense LDL particles [50]. The rise in TAG-rich lipoprotein particles seen in metabolic disorders (e.g., insulin resistance, type II diabetes) results predominantly from the overproduction of apoB100-containing VLDL by the liver, competition of VLDL and CM for binding to lipoprotein lipase in peripheral tissues, and inefficient uptake of TAG-rich lipoprotein remnants by hepatic LDLR [51,52]. Thus, it is critically important to identify new therapeutic agents and strategies that would lessen hypertriglyceridemia and also be safe and relatively inexpensive to implement.

We, and others, reported that LA and PBA possess TAG-rich lipoprotein-lowering properties in animal and cell models [17,18,22,39]. We postulated that the molecular mechanisms by which LA and PBA impart their lipid-lowering properties differ [40]. In this study, we explored the molecular mechanisms exerted by LA and PBA to impede TAG-rich lipoprotein overproduction. We first examined the expression of genes involved in lipolysis, lipogenesis, TAG synthesis, and lipoprotein assembly in HepG2 cells. Both LA and PBA significantly decreased *SREBP1* and *FASN* expression and increased *CPT1A* and *INSIG2*. To our knowledge, this is the first report showing that PBA upregulates genes that contribute to lowering cellular neutral lipids (*CPT1A* and *INSIG2*) while concomitantly repressing lipogenic genes (*SREBP1* and *FASN*). With regards to TAG synthesis and lipidation of VLDL, LA significantly decreased expression of *DGAT1* and *DGAT2*, which catalyze TAG synthesis from diacylglycerol and FFAs, and *MTP*, which transfers TAG to nascent apoB to facilitate lipoprotein secretion. In contrast, PBA did not change the transcript levels of *DGAT1*, *DGAT2* and *MTP*; an observation that is consistent with our earlier work showing PBA decreased secretion of apoB-containing TAG-rich lipoproteins from HepG2 cells without decreasing cellular TAG concentration [40].

As a second messenger, cAMP participates in numerous signaling pathways among which pathways relevant to glucose and lipid metabolism [53]. cAMP stimulates the transcription of lipolytic genes through activation of protein kinase A (PKA) and its substrate, the transcription factor cAMP-response element-binding protein (CREB) [54,55]. cAMP also inhibits lipid synthesis via stimulation of AMP-activated protein kinase (AMPK), which represses mTORC1 activity and SREBP1c-mediated transcription of lipogenic genes (*ACC*, *FASN*, and *SCD*) [56,57], and enhances CPT1α-mediated β-oxidation of long-chain fatty acids [58]. By showing that the inhibition of cAMP production suppressed *CPT1A* transcription in HepG2 cells, our results are consistent with earlier reports of cAMP inducing *CPT1A* mRNA in primary rat hepatocytes [59]. The use of SQ22536, an inhibitor of adenylyl cyclase, revealed that LA’s lipid-lowering properties in liver cells are in part mediated by cAMP. However, the primary target of LA leading to cAMP remains to be identified, as one of the most potent redox couples, LA(S-S)/LA(SH)_2_ is anticipated to interact with extracellular as well as intracellular proteins, primarily those exposed to the oxidized cytoplasmic regions where local concentrations of oxidants (e.g., hydrogen peroxide) build up. In contrast to LA, cAMP did not take part in the mechanism of action of PBA.

The use of SQ22536 also revealed another facet of cAMP-mediated regulation of lipid metabolism; cAMP via CREB contributes to lipogenesis and adipogenesis. Fox et al. showed that the constitutive activation of CREB was sufficient to drive adipogenesis in 3T3-L1 cells, whereas a dominant-negative CREB mutant inhibited this process [60]. Mechanistically, CREB promotes the expression of both PPARγ and C/EBPβ, which are required and sufficient inducers of adipogenesis [61,62]. Here, we provide evidence that the direct blockage of cAMP production leads to a reduction of *FASN* and *DGAT2* transcription. Our results are fully supported by other findings: (i) Antony et al. showed that mRNA levels of *Fasn* were markedly decreased in the lungs of *Creb1 ^−/−^* mice [63], (ii) Erion et al. reported a significant decrease of *Dgat2* mRNAs in ZDF rat livers following knockdown of *Creb1* [64]. Given the dual, seemingly opposite, role of cAMP in lipid metabolism, therapeutic strategies aimed at stimulating cAMP production sustainably may reveal ineffective to control blood lipids and adiposity. In this regard, LA has not been shown to stimulate lipogenesis nor adipogenesis; to the contrary, LA lowers neutral blood lipids and adiposity [16,65,66]. Therefore, it suggests that LA also acts independently of cAMP/PKA.

We previously showed PBA downregulated the secretion of apoB-containing lipoproteins independently of acting as chemical chaperone to relieve ER stress in high glucose-treated HepG2 cells [40]. In the present study, we report that PBA stimulates the acetylation of histones H3 and H4, and histone-associated CBP-p300 to drive *CPT1A* and *INSIG2* expression. Histone acetylation/deacetylation events are essential processes of gene regulation. Acetylation of histone tails by histone acetyltransferases (HAT) relaxes chromatin into a transcription-competent state that facilitates the DNA binding of transcription factors, whereas histone hypoacetylation catalyzed by histone deacetylases (HDAC) is associated with gene silencing [67]. A critical link between lipogenesis, acetyl-CoA availability and histone acetylation was revealed in mammalian cells lacking the acetyl-CoA producing enzyme ATP-citrate synthase (*Acly*) [68]. In that study, Wellen et al. showed that *Acly* knockdown correlated with a decrease in acetylated histones H3 and H4, a decrease in transcription of genes involved in glucose uptake and metabolism, and a decrease in 3T3-L1 adipocyte neutral lipid content. A link between histone acetylation and lipogenesis was further established by Feng et al. [69], who showed that hepatic de novo lipogenesis was markedly increased in mice lacking *Hdac3* suggesting histone hypoacetylation suppresses lipogenesis; conversely histone acetylation stimulates lipogenic gene expression. Unlike *Hdac3* depletion, which caused upregulation of lipogenic genes, our study shows that PBA (an inhibitor of class I and II HDACs; HDAC3 belongs to class I HDACs) selectively upregulated lipid-lowering genes (*CPT1A* and *INSIG2*) while downregulating lipogenic genes (*SREBP1* and *FASN*). PBA’s selectivity may lie in the transcription factors and transcriptional co-activator proteins it regulates, including *PPARα* [70], *PPARγ* [71] and CBP/p300.

We found that constitutively active mTORC1 was associated with the upregulation of *LDLR* and *PCSK9* in HepG2 cells. These changes in gene expression did not translate into significantly elevated levels of LDLR or PCSK9 protein, although PCSK9 trended up under constitutive mTORC1 activation (HepG2-shTSC2 cells). This is not to say that mTORC1 and LDLR are not linked. Liu et al. observed an association between inflammation-mediated activation of mTORC1 and the LDLR pathway in ApoE *^−/−^* mice [72]. They reported a positive correlation between *LDLR* mRNA and p-mTOR (*r* = 0.802, *p* < 0.001, which corroborates our data) but a negative correlation between PCSK9 protein and p-mTOR (*r* = −0.675, *p* < 0.05), suggesting mTORC1 activation induced LDLR expression but repressed that of PCSK9 in ApoE *^−/−^* mice. We did not find this in stably transduced hepatoma cells, suggesting that the mTORC1 signaling and LDLR pathway are connected but PCSK9 is downregulated by the mTORC1 signaling in an ApoE-dependent manner.

A study by Carrier et al. found that LA supplementation increased hepatic LDLR protein two-fold and suppressed plasma PCSK9 by 70% in obese Zucker rats fed a high-fat diet [39]. Consistent with their results, we found LA increased LDLR expression (both mRNA and protein) and lowered PCSK9 secretion in media without changing PCSK9 mRNA or protein expression. PBA markedly enhanced *LDLR* transcription in its own right, but did not change LDLR protein levels. PBA did not affect PCSK9 expression in stable HepG2 cells. Costet et al. showed that SREBP1 promotes PCSK9 gene transcription by tethering to a sterol response element (SRE) in the PCSK9 promoter [73]. Although LA and PBA repressed SREBP1 transcription in HepG2-shTSC2 cells, it did not lower PCSK9 mRNA levels. Evidence of PBA’s therapeutic potential against familial hypercholesterolemia caused by deleterious mutations in *LDLR* was presented by Tveten et al. [74]. LDLR mutants are functional because mutations do not affect the active site but they fail to localize to the plasma membrane and thus accumulate in the ER. In Chinese hamster ovary (CHO) cells expressing the LDLR (G544V) mutant, one of the predominant mutations in familial hypercholesterolemia, PBA (5 mM, 24 h) increased protein expression of the LDLR mutant on the plasma membrane and restored some LDL endocytic activity.

**Figure 7:**
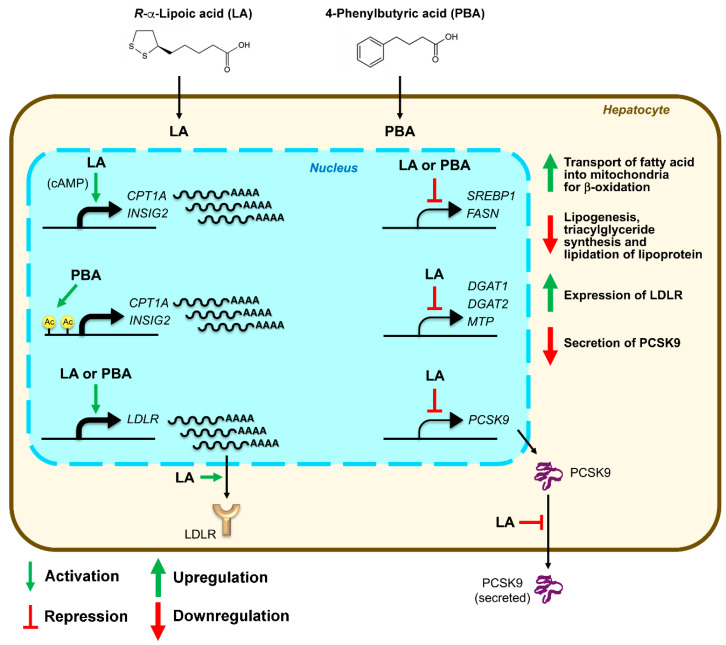
Schematic diagram summarizing the molecular effects of LA and PBA, and expected consequences on lipid metabolism in hepatocytes.

We conclude that (i) both LA and PBA downregulated de novo lipogenesis in an INSIG2/SREBP1-dependent manner, (ii) both LA and PBA activated *CPT1A*, (iii) LA downregulated glycerolipid synthesis genes (*DGAT1* and *DGAT2*) and microsomal triglyceride transfer protein (MTP) whereas PBA did not, (iv) LA upregulated LDLR receptor abundance through downregulation of PCSK9 secretion whereas PBA did not, (v) PBA induced *CPT1A* and *INSIG2* expression through epigenetic acetylation of CBP-p300 at the *CPT1A* and *INSIG2* promoters (Figure 7). Importantly, the lipid-lowering functions of LA and PBA operated under mTORC1 hyperactivation; that is, under conditions needing lipid rebalancing. These results warrant further animal and human studies.

Most animal studies have indicated that LA has hypotriglyceridemic effects, which manifest by a lowering of blood and liver TAG content, and abdominal fat mass. By comparison, research on the TAG-lowering properties of LA in human subjects remains limited. After reviewing the available evidence, it appears that LA is most efficacious (i.e., decreases blood TG by 20–50%) in situations where blood TG is elevated above 200 mg/dL (~2.4 mM) pre-treatment [75,76,77]. In future studies, it will be critically important to consider factors that optimize LA blood plasma levels including the use of bioavailable forms of LA (R-enantiomer, physiologically appropriate salts), the route and timing of administration, as well as the subject characteristics such as the type and severity of hyperlipidemia, other health pre-conditions, gender, age, eating pattern, and lifestyle habits.

## Supplementary Materials

The following are available online at https://www.mdpi.com/2227-9059/8/8/289/s1, Table S1: Oligonucleotide primer sequences used for qRT-PCR, Table S2: Oligonucleotide primer sequences used for ChIP.
