# Supplementary material for: R-α-Lipoic Acid and 4-Phenylbutyric Acid Have Distinct Hypolipidemic Mechanisms in Hepatic Cells
Source: Biomedicines. 2020 Aug 15;8(8):289. doi: 10.3390/biomedicines8080289 (PMC7460023; doi:10.3390/biomedicines8080289)
Supplement: Supplementary file 1 [file biomedicines-08-00289-s001.pdf]

Supplementary Materials

R-*α*-Lipoic Acid and 4-Phenylbutyric Acid Have Distinct Hypolipidemic Mechanisms in Hepatic Cells

Bo He [1] and Régis Moreau [1,*]

[1] Department of Nutrition & Health Sciences, University of Nebraska-Lincoln, Lincoln NE 68583-0806, USA; bhe@huskers.unl.edu (B.H.)
* Correspondence: rmoreau2@unl.edu (R.M.)

List of supplementary materials includes:

Table S1

Table S2

Table S1. Oligonucleotide primer sequences used for qRT-PCR.

Gene	Sequence (5′→3′)[a]	Product size (bp)	GenBank[b]	Ref.
CPT1A	TGGAGTCCGAAATGCTGCAA<br>ACTGACTGTCTTCATCCGATCC	176	NM_001163034.1	c
SREBP1	CAGCCCCACTTCATCAAGG<br>ACTGTTGCCAAGATGGTTCCG	161	NM_004176.4	[1]
MTTP	TGTGGCCTTACTATGGAGGAA<br>AAGGAGCGTAGGTCTTTGCAG	184	NM_000253.2	[2]
PPIA	TTCATCTGCACTGCCAAGAC<br>TGTCCACAGTCAGCAATGGT	152	NM_001300981.1	c
FASN	ACAGCGGGGAATGGGTACT<br>GACTGGTACAACGAGCGGAT	188	NM_004104.4	c
DGAT1	GTTATTGCGGCCAATGTCTT<br>AACCAGTAAGACCACAGCCG	150	NM_012079.5	c
DGAT2	CAGGTCATCTCAGTGCTCCA<br>TCCAGTCAAACACCAGCCAA	139	NM_032564.4	c
INSIG2	CACGGCTTCAGCTGTGATT<br>TCCACTTTAGCACTGGCATG	144	NM_016133.3	c
LDLR	AATGGCATCACCCTAGATCTC<br>CTCCAAGATGGTCTTCCGGTT	111	NM_000527.4	[3]
PCSK9	CCAAGATCCTGCATGTCTTCC<br>AACTTCAAGGCCAGCTCCAG	85	NM_174936.3	[4]

[a] Shown as sense primer followed by antisense primer

[b] GenBank accession number

[c] This study

References

1. Ren, S.; Li, X.; Rodriguez-Agudo, D.; Gil, G.; Hylemon, P.; Pandak, W. M., Sulfated oxysterol, 25HC3S, is a potent regulator of lipid metabolism in human hepatocytes. *Biochem Bioph Res Co* **2007,** 360, 802-808.
2. Dougan, S. K.; Rava, P.; Hussain, M. M.; Blumberg, R. S., MTP regulated by an alternate promoter is essential for NKT cell development. *Journal of Experimental Medicine* **2007,** 204, 533-545.
3. Tveten, K.; Ranheim, T.; Berge, K. E.; Leren, T. P.; Kulseth, M. A., Analysis of alternatively spliced isoforms of human LDL receptor mRNA. *Clin Chim Acta* **2006,** 373, (1-2), 151-7.
4. Nilsson, L. M.; Abrahamsson, A.; Sahlin, S.; Gustafsson, U.; Angelin, B.; Parini, P.; Einarsson, C., Bile acids and lipoprotein metabolism: effects of cholestyramine and chenodeoxycholic acid on human hepatic mRNA expression. *Biochem Biophys Res Commun* **2007,** 357, (3), 707-11.

Table S2. Oligonucleotide primer sequences used for ChIP.

Gene	Sequence (5′→3′)[a]	Product size (bp)	GenBank[b]	Ref.
CPT1A	GACAGCGGGAGAAATGCAAC<br>AGTGAGCCCCTTTTAGACGC	171	NM_001163034.1	c
INSIG2	AGGGGAGGAAAAGGAGCAAA<br>TGTGGGCGGAGTAAAGGAAT	168	NM_016133.3	c

[a] Shown as sense primer followed by antisense primer

[b] GenBank accession number

[c] This study
